# Diabetes and Pre-Diabetes among Persons Aged 35 to 60 Years in Eastern Uganda: Prevalence and Associated Factors

**DOI:** 10.1371/journal.pone.0072554

**Published:** 2013-08-14

**Authors:** Roy William Mayega, David Guwatudde, Fredrick Makumbi, Frederick Nelson Nakwagala, Stefan Peterson, Goran Tomson, Claes-Goran Ostenson

**Affiliations:** 1 Division of Global Health (IHCAR), Department of Public Health Sciences, Karolinska Institutet, Stockholm, Sweden; 2 Department of Epidemiology and Biostatistics, Makerere University School of Public Health, Kampala, Uganda; 3 Department of Internal Medicine, Mulago National Referral Hospital, Kampala, Uganda; 4 International Maternal and Child Health Unit, Uppsala University, Uppsala, Sweden; 5 Division of Medical Management Centre (MMC), Department of Learning, Informatics, Management and Ethics (LIME), Karolinska Institutet, Stockholm, Sweden; 6 Endocrine and Diabetes Unit, Department of Molecular Medicine and Surgery, Karolinska Institutet, Stockholm, Sweden; 7 Department of Health Policy, Planning and Management, Makerere University School of Public Health, Kampala, Uganda; German Diabetes Center, Leibniz Center for Diabetes Research at Heinrich Heine University Duesseldorf, Germany

## Abstract

**Background:**

Our aim was to estimate the prevalence of abnormal glucose regulation (AGR) (i.e. diabetes and pre-diabetes) and its associated factors among people aged 35-60 years so as to clarify the relevance of targeted screening in rural Africa.

**Methods:**

A population-based survey of 1,497 people (786 women and 711 men) aged 35-60 years was conducted in a predominantly rural Demographic Surveillance Site in eastern Uganda. Participants responded to a lifestyle questionnaire, following which their Body Mass Index (BMI) and Blood Pressure (BP) were measured. Fasting plasma glucose (FPG) was measured from capillary blood using On-Call® Plus (Acon) rapid glucose meters, following overnight fasting. AGR was defined as FPG ≥6.1mmol L^-1^ (World Health Organization (WHO) criteria or ≥5.6mmol L^-1^ (American Diabetes Association (ADA) criteria. Diabetes was defined as FPG >6.9mmol L^-1^, or being on diabetes treatment.

**Results:**

The mean age of participants was 45 years for men and 44 for women. Prevalence of diabetes was 7.4% (95%CI 6.1-8.8), while prevalence of pre-diabetes was 8.6% (95%CI 7.3-10.2) using WHO criteria and 20.2% (95%CI 17.5-22.9) with ADA criteria. Using WHO cut-offs, the prevalence of AGR was 2 times higher among obese persons compared with normal BMI persons (Adjusted Prevalence Rate Ratio (APRR) 1.9, 95%CI 1.3-2.8). Occupation as a mechanic, achieving the WHO recommended physical activity threshold, and higher dietary diversity were associated with lower likelihood of AGR (APRR 0.6, 95%CI 0.4-0.9; APRR 0.6, 95%CI 0.4-0.8; APRR 0.5, 95%CI 0.3-0.9 respectively). The direct medical cost of detecting one person with AGR was two US dollars with ADA and three point seven dollars with WHO cut-offs.

**Conclusions:**

There is a high prevalence of AGR among people aged 35-60 years in this setting. Screening for high risk persons and targeted health education to address obesity, insufficient physical activity and non-diverse diets are necessary.

## Introduction

Type 2 diabetes is rising in sub-Saharan Africa (SSA) [1,2]. Between 2010 and 2030, a 69% increase in type 2 diabetes is expected among adults aged 20-79 years in low-income countries compared with a 20% increase in the same age group in high income countries [3]. This increase will strain health systems already facing a high burden of communicable diseases [1,4], and in which non-communicable disease (NCD) interventions at primary care levels are almost non-existent.

Despite the rising burden of type 2 diabetes in SSA, data on its epidemiology are scarce, yet such evidence is necessary for planning interventions. Systematic reviews show that the prevalence of diabetes in Africa varies between countries and rural-urban gradients, from 0.6% (among people aged 13 years and over) in rural Uganda to 12% (among people aged 17 years and over) in urban Kenya [5–8]. However, these studies included a wide age-range of participants and therefore do not sufficiently clarify the prevalence of type 2 diabetes in people older than 30 years, who are at higher risk of type 2 diabetes. In Uganda information on type 2 diabetes prevalence is from a few locale-specific surveys due to lack of national NCD survey data [5]. A crossectional study in Kampala and Mukono Districts in 2002 estimated the prevalence of type 2 diabetes to be 8% among people aged ≥35 years [9]. However, these districts have a mainly urban population. Another survey in rural southern Uganda estimated the prevalence of diabetes at 0.6% among people aged ≥13 years [8]. That survey, however, did not provide age-specific prevalences, yet it included a wide age range. Moreover, they used random blood sugar to assess abnormal glucose regulation (AGR).

Most type 2 diabetes patients go through a ‘pre-diabetes’ phase for several years [10,11], during which there is an opportunity to identify them and initiate timely prevention. Pre-diabetes is defined as a fasting plasma glucose (FPG) level of 6.1-6.9mmol L^-1^ or 5.6-6.9mmol L^-1^ by different criteria (Impaired Fasting Glucose (IFG)) [12,13], or from an Oral Glucose Tolerance Test (OGTT) as a 2-hour post-load plasma glucose level of 7.8-11mmol L^-1^ (Impaired Glucose Tolerance (IGT)) [12], or a Haemoglobin A_1C_ level of 5.7-6.4% [13].

Effective prevention requires risk stratification [14], and persons with pre-diabetes represent a high risk group for type 2 diabetes [15–18]. There is insufficient data on the prevalence of pre-diabetes in SSA, more importantly in persons older than 30 years.

Without prevalence data, it is difficult to justify targeted screening for high risk persons [11,19]. The percentage of the population requiring long-term care and the cost of detecting one high risk person are also unclear. The objective of this study was to estimate the prevalence of type 2 diabetes and pre-diabetes among people aged 35-60 years (determined by FPG), the associated socio-behavioural factors, and the cost of detecting one high risk person, so as to clarify whether targeted screening for this age-group is justified in a rural low-income setting.

## Methods

### Setting

The study was conducted in the Iganga-Mayuge Health and Demographic Surveillance Site (HDSS) [20] in Eastern Uganda. The HDSS has a population of approximately 70,000 residing in 65 villages, of which 13 villages are peri-urban and 52 are rural. Routine data are collected on births, deaths, and in- and out- migrations. Add-on studies are also often conducted within the HDSS, including our study. Data for this study were collected in March and April 2012.

### Study Population

This study was nested into a larger survey that assessed the prevalence of overweight and hypertension [21]. The study population comprised adult men and women aged 35-60 years within the HDSS. A multi-stage design was used: Forty-two villages (8 peri-urban and 34 rural) were selected, from which a simple random sample of 1,656 participants was taken proportionate to the village population sizes, using the HDSS database and Microsoft Excel®. HDSS locator information was used to trace participants to their households.

### Measurements

Participants underwent physical measurements (height, weight, and blood pressure (BP), and a plasma glucose test) and responded to a questionnaire that assessed socio-behavioural characteristics. Data were collected by 12 teams of two experienced research assistants each (a nurse and a social worker). The teams underwent five days of training.

To assess FPG, each participant was contacted a day before their scheduled date for data collection and an appointment was sought for the following day. They were requested not to eat anything after their evening meal until their blood test had been conducted the following morning. All appointments were set in the morning hours before 10.30am. Data collectors covered only four households per team per-day. For participants who reported having eaten anything before the blood test, a new appointment was requested.

Each consenting participant underwent an FPG test. One drop of capillary blood obtained from a finger prick (using an automated lancing device) was placed on an applicator and analysed using a simple automated glucometer (On-Call® Plus, Acon). ‘Point-of-Care’ (POC) tests are widely recommended for self monitoring of blood glucose [22,23]. Because of their simple procedure, they do not require special laboratory skills. The tests were therefore performed by the nurses on the data collection teams. From the product’s information insert, the Coefficients of Variation (CV) for the On-Call® Plus test are within acceptable range (less than 10%) for the different plasma glucose values in the range tested: at 2.6mmol L^-1^ (SD=0.14mmol L^-1^); at 4.4mmol L^-1^ (CV = 3.3%); at 8.4mmol L^-1^ (CV = 3.0%); at 13.6mmol L^-1^ (CV = 3.1%); and at 21.1mmol L^-1^ (CV = 2.4%).

Classification of abnormal glucose regulation (AGR) was based on the revised World Health Organization (WHO) criteria [12] and the American Diabetes Association (ADA) criteria [13]: An FPG <6.1mmol L^-1^ (WHO criteria) or <5.6mmol L^-1^ (ADA criteria) for a participant not on diabetes treatment was classified as normal. ‘Abnormal Glucose Regulation’ (AGR) was defined as an FPG ≥6.1mmol L^-1^ (WHO criteria) or ≥5.6mmol L^-1^ (ADA criteria), or being on diabetes treatment regardless of glycaemic status [12,13]. AGR included two categories: 1) Those with diabetes (participants with an FPG≥7mmol L^-1^, or on diabetes treatment regardless of glycaemic status) and, 2) those with pre-diabetes (participants with an FPG between 6.1-6.9mmol L^-1^ (WHO criteria) or 5.6-6.9 (ADA criteria) and not on diabetes treatment).

Procedures used to measure socio-behavioural characteristics have already been described in detail elsewhere [21]. Height was measured using standard height meters, with the participant standing upright. Weight was measured using calibrated Seca® scales, with the participant lightly clothed. Body Mass Index (BMI) was then calculated as weight-in-kilograms divided by the square of height-in-metres. Participants were classified as underweight if their BMI was <18.5 Kgm^-2^, normal weight if their BMI was 18.5-24.9, overweight if their BMI was 25.0-29.9 and obese if their BMI was ≥30 Kgm^-2^ [24]. Two BP measurements were taken (5 to 30 minutes apart), with the participant seated, using calibrated electronic devices (Welch-Allyn®). The mean of these two measurements was calculated as their BP. Participants were classified as hypertensive if their systolic BP was ≥140mmHg, or if their diastolic BP was ≥90mmHg, or if they were on anti-hypertensive drugs [25].

A structured questionnaire was used to collect data on socio-behavioural factors, including demographic characteristics, family history of diabetes, physical activity, tobacco and alcohol use, and food frequency. The questionnaire was translated into the local language and pre-tested in a non-study village. The socio-behavioural questionnaire was adapted from validated tools, especially the WHO STEPs tool [26] which has been used in several African countries for risk factor assessment [27]. Questions on physical activity are derived from the WHO Global Physical Activity Questionnaire (GPAQ), shown to have a high reliability (kappa of 0.67-0.93 in various studies) [28,29]. The GPAQ has also been evaluated in Africa, showing acceptable validity [30]. Questions on alcohol use and smoking were shown in studies by the WHO to have good reliability for assessing dependency (Coefficients of 0.7-0.9) [31,32]. The individual dietary diversity score used in our study has been shown to have high validity in assessing food quality (coefficients of 0.33 to 0.97 in various studies, some in Africa) [33,34].

To classify socio-economic status (SES), principal components analysis (PCA) was done on 11 household assets: 1) a radio, 2) a television, 3) a mobile phone, 4) a bicycle, 5) a motorcycle, 6) a motor vehicle, 7) a piece of land, 8) large farm animals like cattle, goats and sheep, 9) small farm animals like poultry, 10) a manufactured bed, and 11) a brick and cement house. The component on which most assets loaded was used to generate an SES score for each participant. Participants’ SES scores were then grouped into SES tertiles. This approach is used in Demographic Surveillance Surveys in Africa [35].

Questions on physical activity sought information on participants’ undertaking of ‘vigorous-intensity activities’ (e.g. lifting heavy loads, digging, construction work, etc) and ‘moderate-intensity activities’ (e.g. brisk walking, carrying light loads, riding a bicycle, light recreational activities, etc). Time spent on these activities in a typical week was recorded. Participants were classified into those that met the WHO minimum recommendations for physical activity (at least 75 minutes of vigorous-intensity, or 150 minutes of moderate-intensity activities per week) and those that did not [36].

Tobacco use was assessed using questions on current or ever use of tobacco, whereas alcohol use was assessed with questions on frequency, type and quantity of alcohol consumed. Participants were classified as engaging in ‘harmful alcohol use’ if they consumed the equivalent of at least 60 grams of pure alcohol either regularly or in a binge in the 1 month preceding the survey [26,37]. Information was also collected on family history of diabetes, defined as a history of diabetes in one 1^st^ degree or at least two 2^nd^ degree relatives [38].

Food frequency was assessed using recall of foods eaten in the seven days preceding the survey. Local foods were grouped into nine food groups recommended for use in dietary diversity assessments [39], namely: 1) cereals 2) tubers and plantations 3) pulses, 4) vegetables, 5) fruits, 6) milk and dairy products, 7) meats, offal and poultry, 8) fish, and 9) oil/fat. Individual dietary diversity was then assessed by giving a score of “1” to a food group if the participant had eaten at least one food in that group, or a “0” if they did not eat any food in that group [39]. For example, a participant reporting to have eaten sweet potatoes at least once in the previous week would score a “1” under the ‘tubers/plantains’ food group, and a participant who did not eat any food listed in this group would score a “0” for this food group. Since there were nine food groups, the maximum possible score was nine. A score of 0-3 was regarded as ‘low dietary diversity’, 4-6 as ‘moderate diversity’ and 7-9 as ‘high diversity’ [39].

To estimate the cost of screening all people aged 35-60 years, the additional direct costs of implementing such a program were computed and annualised for one round of health facility-based screening. A health services perspective has been used, focusing on direct medical costs. The main assumption is that all people aged 35-60 years in the district (i.e. 13.5% of an estimated population of 466,200 [40]) are offered an FPG test once over a two year period (hence 31,469 per year) at any visit to a public health facility with laboratory services. The two year period spreads out the costs from salaries and health worker time, which are not included in this analysis. The capital costs include: 1) The price of hand-held Glucometers (Obtained from the product catalogue of the national procuring agency for pharmaceuticals in Uganda) – (one each for the 12 health facilities that offer laboratory services in Iganga district, assuming each Glucometer lasts three years); 2) The cost of training health workers (two each for 12 health centres, and assuming that re-training occurs after five years). The recurrent costs include: 1) Costs of the FPG test strips (assuming a wastage rate of 2% as found in this study), and 2) the costs of batteries to power the Glucometers.

### Statistical analysis strategy

Data were double entered in EpiData, cleaned and exported to STATA10 for analysis.

The prevalence of diabetes and pre-diabetes was calculated as the total number of people with diabetes or pre-diabetes respectively divided by the total study sample, presented as a percentage. To determine association between AGR and socio-behavioural factors, we used Prevalence Rate Ratios (PRR) rather than Odds Ratios because the independent variable (AGR) was highly prevalent. Odds Ratios tend to over-estimate the strength of association in such scenarios [41,42]. PRRs were estimated using the Modified Poisson regression analysis model, with robust standard errors [42]. The independent factors included in the adjusted analysis were: demographic variables (all factors with a p<0.1 at bivariate analysis), family history of diabetes, BMI level, BP level, physical activity level, tobacco use, alcohol use and dietary diversity. We present unadjusted and adjusted PRRs, plus their confidence intervals (CI) and p-values at α=0.05.

To estimate the cost of detecting one person with AGR, the estimated capital costs of such a program, scaled to the district level, were annualized and added to the estimated recurrent costs for one year to obtain the total annual cost. The cost per person screened was computed. The number of people with AGR that would be detected in one round of a health facility based district-wide screening program targeting people aged 35-60 years was computed based on the prevalence of AGR in this age-group. Thereafter, the cost of detecting one person with AGR (based on WHO and ADA cut-offs) was computed. Using prevalence data for overweight, we also assessed the cost of detecting one person with AGR if screening is targeted only to overweight persons. The costs presented are for one round of screening.

### Ethics statement

This study was approved by Makerere University School of Public Health Higher Degrees Research and Ethics Committee (30^th^ June 2011), the Swedish Regional Ethics Board (Stockholm) (Diary Number 2010/2049-31/2) and the Uganda National Council of Science and Technology. Permission was also obtained from the Iganga-Mayuge HDSS management. Each participant signed a full informed consent form.

## Results

### Background characteristics of respondents

Table 1 summarizes the background characteristics of respondents. Of 1,656 eligible participants contacted, 1,497 (90.4%) participated in the study, 786 of whom were women (52.5%) and 711 were men (47.5%). Reasons for non-participation included declining the blood test (4.2%), and being away from one’s residence at three consecutive visits (5.4%). The mean age of respondents was 44.5 years for men, 43.7 years for women, and 44 years overall (Standard Deviation (SD) = 6.9). The age distribution of our study sample and that for 35-60 year-olds in the HDSS were similar. Sixty-two percent of participants were subsistence farmers. Nineteen percent of participants had not received formal education. A family history of diabetes was reported in 12.2% of participants (Table 1).

**Table 1:** Distribution of background and socio-behavioural characteristics of respondents by glucose regulation status.

**Diabetes/Prediabetes Status^a^ (%)**	**Normal **	**Have prediabetes**	**Have diabetes**	**Total -n-(%)**	**95% CI**
**Background Factors**					
Sex:					
Males	84.2	9.3	6.5	711 (47.5)	[44.7-50.3]
Females	83.8	8.0	8.1	786 (52.5)	[49.7-55.3]
Location of residence:					
Rural	83.7	9.5	6.8	1275 (85.2)	[79.7-89.4]
Peri-urban	86.0	3.6	10.4	222 (14.8)	[10.7-20.3]
Age-group:					
35-39	86.2	8.1	5.3	456 (30.5)	[25.4-36.2]
40-44	85.1	7.9	7.0	357 (23.8)	[22.1-25.5]
45-49	82.4	10.5	7.1	324 (21.7)	[19.0-24.6]
50-54	80.3	8.2	11.5	208 (13.9)	[10.3-18.6]
55-60	82.2	8.6	9.2	152 (10.2)	[08.9-11.6]
Main occupation:					
Mechanic	87.6	5.7	6.8	177 (11.8)	[0.92-15.0]
Trader	81.3	9.7	9.0	289 (19.3)	[16.1-23.0]
Formal/Salaried	79.6	9.2	11.2	98 (6.6)	[04.8-09.0]
Subsistence farmer	84.7	8.8	6.5	933 (62.3)	[58.8-65.7]
Highest level of education:					
None	81.4	12.4	6.2	291 (19.4)	[15.1-24.7]
Lower Primary	84.4	7.9	7.6	315 (21.0)	[20.1-22.0]
Higher Primary	85.2	7.2	7.6	539 (36.0)	[32.7-39.5]
Secondary	85.1	8.0	6.9	275 (18.4)	[17.2-19.7]
Tertiary	80.5	9.1	10.4	77 (5.1)	[04.0-06.6]
Family history of diabetes:					
No	84.7	8.4	6.8	1315 (87.8)	[83.2-91.3]
Yes	79.1	9.9	11.0	182 (12.2)	[08.7-16.8]
SES Tertile					
Lowest	82.6	9.7	7.7	495 (33.1)	[30.0-36.3]
Middle	86.5	7.6	6.0	503 (33.6)	[28.5-39.1]
Highest	83.0	8.6	8.4	499 (33.3)	[30.7-36.0]
BMI					
Normal	84.5	9.0	6.5	988 (66.0)	[62.2-69.6]
Under-weight	90.0	6.7	3.3	240 (16.0)	[10.7-23.4]
Overweight	80.4	8.5	11.1	189 (12.6)	[09.5-16.5]
Obese	68.8	10.0	21.3	80 (5.3)	[01.8-15.2]
Hypertensive					
No	83.8	9.2	7.1	1190 (79.5)	[76.6-82.1]
Yes	85.0	6.5	8.5	307 (20.5)	[17.9-23.4]
**Socio-behavioural factors**					
Attains sufficient physical activity					
No	77.1	10.6	12.4	218 (14.6)	[09.0-22.7]
Yes	85.2	8.3	6.5	1279 (85.4)	[77.3-91.0]
Dietary diversity					
Low	80.3	9.6	10.2	324 (21.6)	[16.6-27.7]
Moderate	84.4	8.7	6.9	1,021 (68.2)	[65.9-70.5]
High	84.5	5.9	4.6	152 (10.2)	[07.1-14.3]
Current tobacco user					
No	84.2	8.6	7.2	1409 (94.1)	[93.2-95.0]
Yes	81.8	9.1	9.1	88 (5.9)	[05.1-06.8]
Harmful alcohol user					
No	83.7	8.6	7.6	1427 (95.3)	[92.8-97.0]
Yes	85.8	8.5	5.7	70 (4.7)	[03.0-07.2]
**All (WHO Criteria)**	**84.0**	**8.6**	**7.4**	**1497 (100.0)**	
**All (ADA Criteria)**	**72.5**	**20.2**	**7.4**	**1497 (100.0)**	

WHO = World Health Organization; ADA = American Diabetes Association

^a^Based on World Health Organization criteria for classification of Abnormal Glucose Regulation, and whether a participant was already on diabetes treatment or not

### Prevalence of diabetes and pre-diabetes

**Figure 1:**
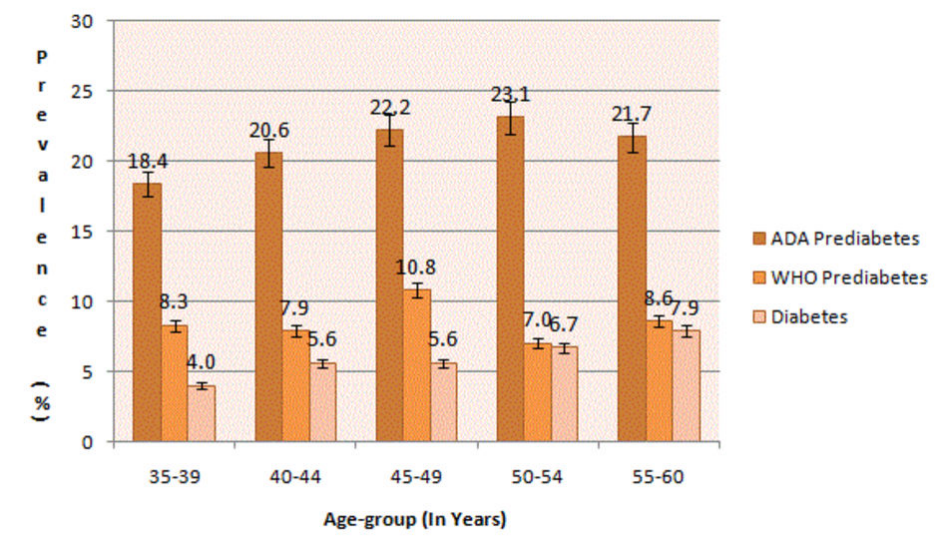
Prevalence of abnormal glucose regulation by age category.

Table 1 shows findings on prevalence of diabetes and pre-diabetes. The mean FPG value for participants was 5.3mmol L^-1^ (Range 2.5-29.9mmol L^-1^; SD=1.6). The prevalence of diabetes was 7.4% (95%CI 6.1-8.8). This included 82 participants with FPG≥7mmol L^-1^ (5.5%) and 28 on diabetes treatment but with FPG <7.0mmol L^-1^ (1.9%). Using WHO criteria, the prevalence of pre-diabetes was 8.6% (95%CI 7.3-10.2). The ADA cut-offs resulted in a higher prevalence of pre-diabetes (20.2%; 95%CI 17.5-22.9). Prevalence of diabetes and the ratio of diabetes: pre-diabetes increases with increasing 5 year age-categories within the age-group studied but prevalence of AGR levels-off from 50–60 years (Figure 1).

### Knowledge about own diabetes status

Among the 82 participants with FPG levels in the range classified as diabetes, only 16 (19.5%) were aware that they had diabetes. Overall, only 44 of the 110 participants (40%) who had diabetes (either an FPG level in the diabetes range or were on diabetes treatment), knew that they had diabetes. Among 44 participants on diabetes treatment, only 28 (63.6%) had their glycaemia controlled (i.e. FPG<6.9 mmol L^-1^)

### Factors associated with abnormal glucose regulation

Table 2 shows factors associated with AGR among people aged 35-60 years. The prevalence of AGR was 2 times higher among obese persons compared with participants who had a normal BMI (APRR 1.9, 95% CI 1.3-2.8). However, prevalence of AGR did not differ significantly between overweight participants and those with a normal BMI (APRR1.2, 95%CI 0.8-1.7). Persons who met the WHO minimum recommended physical activity level and those who had a higher dietary diversity score had a significantly lower likelihood of AGR (APRR 0.6, 95%CI 0.4-0.8; APRR 0.5, 95%CI 0.3-0.9 respectively) (Table 2).

**Table 2:** Factors associated with abnormal glucose regulation among people aged 35-60 years.

**Factors**	**Category**	**-n-**	**% with abnormal glucose regulation^a^**	**Un-adjusted**	**Adjusted**†	**p-value**	**Adjusted, intervening variables excluded^b^**	**p-value**
			**(%)**	**PRR [95%CI]**	**APRR [95%CI]**		**APRR [95%CI]**	
**Background factors**								
Sex	Male	711	14.4	1.0				
	Female	786	14.5	1.0 [0.79-1.29]				
Age group	35-39 years	457	12.3	1.0	1.0		1.0	
	40-49 years	679	14.9	1.2 [0.89-1.64]	1.2 [0.86-1.58]	0.327	1.1 [0.86-1.58]	0.322
	50-60 years	361	16.4	1.3 [0.95-1.87]	1.3 [0.90-1.77]	0.185	1.3 [0.93-1.83]	0.127
Location of residence	Rural	1275	14.7	1.0	1.0		1.0	
	Urban	222	13.1	0.9 [0.61-1.28]	0.6 [0.41-0.88]	0.010	0.8 [0.56-1.20]	0.314
Education level	None	291	17.5	1.0				
	Primary	854	13.4	0.8 [0.56-1.03]				
	Secondary	275	13.5	0.8 [0.52-1.13]				
	Tertiary	77	18.2	1.0 [0.61-1.77]				
Occupation	Subsistence	933	14.5	1.0	1.0		1.0	
	Traders	289	15.9	1.1 [0.81-1.50]	1.0 [0.75-1.37]	0.932	1.1 [0.81-1.51]	0.522
	Formal salaried	98	19.4	1.3 [0.87-2.07]	1.2 [0.79-1.94]	0.357	1.4 [0.91-2.22]	0.126
	Mechanic	177	9.0	0.6 [0.38-1.02]	0.6 [0.37-0.97]	0.038	0.6 [0.39-1.05]	0.078
SES tertile	Lowest	495	15.8	1.0				
	Middle	503	12.5	0.8 [0.58-1.08]				
	Highest	499	15.0	1.0 [0.71-1.28]				
Family History of diabetes	No	1315	13.8	1.0	1.0		1.0	
	Yes	182	19.2	1.4 [1.00-1.94]	1.2 [0.87-1.68]	0.258	1.3 [0.96-1.85]	0.085
BMI	18.5-24.9	988	14.2	1.0	1.0			
	<18.5	240	8.8	0.6 [0.40-0.95]	0.6 [0.40-0.94]	0.027		
	25-29.9	189	17.5	1.2 [0.87-1.74]	1.2 [0.83-1.67]	0.349		
	30+	80	27.5	1.9 [1.31-2.86]	1.9 [1.30-2.83]	0.001		
Hypertensive	No	1190	14.5	1.0				
	Yes	307	14.0	1.0 [0.71-1.31]				
**Socio-behavioural factors**								
Attains sufficient physical activity	No	218	20.6	1.0	1.0			
	Yes	1279	13.4	0.6 [0.48-0.87]	0.6 [0.44-0.83]	0.002		
Dietary diversity	Low	324	17.9	1.0	1.0		1.0	
	Moderate	1021	14.0	0.8 [0.59-1.03]	0.8 [0.59-1.03]	0.083	0.8 [0.57-1.01]	0.055
	High	152	9.9	0.6 [0.32-0.94]	0.5 [0.32-0.93]	0.026	0.5 [0.32-0.93]	0.025
Current tobacco user	No	1409	14.3	1.0				
	Yes	88	17.1	1.2 [0.74-1.93]				
Harmful alcohol use	No	1427	14.7	1.0				
	Yes	70	10.0	0.7 [0.33-1.39]				

PRR = Unadjusted Prevalence Rate Ratio; APRR = Adjusted Prevalence Rate Ratio († Adjusted for residence, age, occupation, family history of diabetes, BMI, physical activity level and dietary diversity)

^a^Based on World Health Organization criteria

^b^This analysis excludes BMI and Physical activity, which are possible intervening variables in the relationship between occupation and AGR; other models that excluded BMI alone and physical activity alone were also run; however, the change in – 2Log Likelihood values for all models without intervening variables, compared with the original model was way less than 10%, indicating that the mediation effect was not substantial

AGR was significantly lower among persons employed as mechanics compared to subsistence farmers (APRR 0.6, 95%CI 0.3-0.9). However, further analysis shows that although the majority of our study population achieves the recommended physical activity threshold, significantly more mechanics achieve this threshold (91.6%) compared with subsistence farmers (79.9%). Physical activity is therefore an intervening variable in the relationship between occupation and AGR.

Although residence was not significantly associated with AGR at bivariate analysis, adjusting for physical activity resulted in a 40% lower likelihood of AGR among participants from peri-urban villages compared with those from rural villages (APRR 0.6, 95% CI 0.4-0.9). Although participants with adequate physical activity were disproportionately more in the rural than the peri-urban villages, controlling for physical activity results into lower likelihood of AGR in peri-urban than rural dwellers. Physical activity is therefore a confounder of the relationship between place of residence and AGR. There was no significant association between sex, age group, level of education, social economic status, hypertension, tobacco use and harmful alcohol use and AGR.

Analysis was also done for factors associated with diabetes. Findings show that obesity was the only factor significantly associated with having diabetes (Refer to Supplementary table S1).

### Cost per high risk individual discovered through screening

The total annualized direct medical costs of screening all people aged 35-60 years in the district over 2 years, assuming that 12 health centres are involved, was sixteen thousand five hundred and seventy-seven United States Dollars (USD) (Table 3). Based on this, the cost per person screened is zero point five three USD per year. At the current level of prevalence, the cost of detecting one person with AGR among people aged 35-60 years is estimated at three point seven USD when WHO criteria are used and two point zero USD when ADA criteria are used. If screening is targeted to overweight persons only, the cost of detecting one person with AGR in this age-group is estimated at three point zero USD with WHO criteria and one point seven USD with criteria (Table 3).

**Table 3:** Estimated additional direct costs of screening for abnormal glucose regulation (AGR) among 35-60 year-olds.

**Source of cost**	**Number**	**Unit Cost ($)**	**Total Cost ($)**	**Years in use for Capital items**	**Total annualized costs ($)**
**Capital costs**					
Hand-held Glucometers^a^	12	60	720	3	240
Training of Health workers^b^	24	40	960	5	192
**Recurrent costs**					
Tests for 62,937 people (in 2 years)^c^	64196	0.5	32098		16,049
Dry cells for the Glucometers^d^	48	2	96		96
**Total cost**					**16,577**
Number with AGR (WHO criteria) (14.4% of 31,469 screened p.a.)					4,531
Number with AGR (ADA criteria) (26.2% of 31,469 screened p.a.)					8,245
***Costperpersonscreened***					***0.53***
***CostperpersonwithAGRdetected(WHOcriteria)***					***3.7***
***CostperpersonwithAGRdetected(* *ADA* *criteria* *)***					***2.0***
**Among overweight persons only**					
Tests for 11,329 over-weight people^e^	11556	0.5	5778		2,889
**Total cost for overweight persons only**					**3,417**
Number with AGR among overweight people (WHO criteria) (20.4%)					1,156
***CostperpersonwithAGRdetectedamongoverweightpersons(BMI≥25kgm*^*-2*^*)(WHOcriteria)***					***3.0***
Number with AGR among overweight people (ADA criteria) (34.6%)					1,961
***CostperpersonwithAGRdetectedamongoverweightpersons(BMI≥25kgm*^*-2*^*)(* *ADA* *criteria* *)***					***1.7***

WHO = World Health Organization (Cut off for AGR=5.6 mmolL^- 1^ ; ADA = American Diabetes Association (Cut off for AGR=5.6 mmol L^- 1^)

**Assumptions**: This analysis is restricted to ‘additional costs’ of the screening program

^a^That a glucometer lasts about 3 years (based on Iganga Hospital data)

^b^Iganga District has 12 Health Centres that provide laboratory services; that 2 health workers are trained per health centre; that a health worker needs re-training after 5 years.

^c^Iganga District has a population of 466200 of whom 13.5% (62937) are aged 35-60 years; that 35-60 year old persons are screened once in 2 years, hence 31469 people screened per year; the required number of FPG tests is adjusted for 2% wastage rate observed in our study; that every person in the district visits a health centre at least once in 2 years.

^d^That each glucometer needs 4 pairs of batteries per year (based on Iganga Hospital data)

^e^Prevalence of overweight among people aged 35-60 years in our study was 18%

## Discussion

Our study departs from prior studies on prevalence of type-2 diabetes in SSA by assessing its prevalence in the 35-60 year age-group. In contrast to studies that observed a general population sample, we demonstrate that the prevalence of diabetes and pre-diabetes among 35-60 year olds in this predominantly rural area is substantial, and needs attention. We illustrate that the majority of people with diabetes do not know their disease status. From a health services perspective, our study provides preliminary data on the costs of a screening program targeting 35-60 year-olds. We assert that in this predominantly rural setting, abnormal glucose regulation is associated with modifiable risk factors that could be the starting point for primary care interventions.

The prevalence of FPG values in the diabetes defining range among 35-60 year-olds in this predominantly rural setting was substantial, with possible under-estimation because the OGTT was not performed. These findings are in marked contrast with other population based studies in which the prevalence of diabetes in most rural settings in SSA was less than 2% [1,5,7,8,43,44]. The reason for this difference is that earlier studies observed participants from a wide age range (≥13, ≥15, ≥17, and ≥25 years) including younger participants who would likely have lower diabetes prevalence, and therefore do not adequately represent the prevalence in people aged 35-60 years. Studies with population level prevalence of diabetes higher than 5% were performed in urban settings [5,9,43,45] with participants from varied age ranges (≥17, ≥35 years). Affirming what has been observed in other studies, our study shows that the proportion of people with diabetes who know their disease status was very low [1,8,46,47]. The significance of our findings is that screening for early detection of type-2 diabetes among people aged 35-60 years may be necessary in this setting.

Not many studies have estimated the prevalence of IFG in SSA. Our study estimates that 8.6% of people aged 35-60 years have pre-diabetes (using WHO criteria), and many more with ADA criteria (20.2%), with possible under-estimation because the OGTT was not performed. These findings are in contrast to the 3% prevalence of pre-diabetes estimated in rural southern Uganda [8]. The study in southern Uganda however included all age-groups from 13 years and above, and did not provide age-specific prevalences of pre-diabetes. A World Bank publication estimated the prevalence of IGT to vary between 2.2% and 16% in different settings in Africa and notes that ‘the impact of type-2 diabetes is bound to continue if nothing is done to curb the rising prevalence of IGT’ [48]. These findings further support the need for early detection of pre-diabetes in people aged 35-60 years.

The marked contrast in the prevalence of pre-diabetes between the WHO and ADA cut-offs has important implications for screening programs. Using the ADA cut-off would imply a significantly larger number of people that need chronic care compared with using the WHO criteria. The consequence of a lower cut-off for resource constrained countries has been highlighted by the WHO [12]. However, the lower cut-off may be beneficial in detecting as many high risk persons as possible, for timely preventive intervention.

Our findings show that AGR is associated with modifiable risk factors including obesity, occupations with lower physical exertion, low physical activity levels and low dietary diversity. These priority groups need to be targeted for more intense lifestyle education that emphasizes increased physical activity, diverse diets, self monitoring of body weight and periodic fasting blood sugar checks. These activities may be integrated into outpatient departments at primary health care levels. The observation that 14% of participants with a normal BMI had AGR further supports the need for periodic screening of all persons older than 35 years for pre-diabetes.

Obesity was the most important factor found to be independently associated with AGR in people aged 35-60 years. This relationship has been demonstrated in other studies from mostly urban settings in SSA [1,9,12,49], but was negated in Maher’s study in rural southern Uganda, which found no association between BMI and AGR [8]. However, Maher’s study looked at a much wider age-group (≥13 years), and used random blood sugar.

The finding that mechanics had a lower likelihood of AGR than subsistence farmers is explained in additional analysis showing that although the majority of our study population achieves the recommended physical activity threshold, significantly more mechanics achieve the recommended threshold compared to subsistence farmers. The observed association between being a mechanic and lower likelihood of AGR is therefore mediated by attainment of the recommended physical activity threshold. A key question arising from this finding is whether participating in subsistence agriculture implies lower physical exertion. This finding is unexpected and therefore highlights a need for further studies that explore work patterns and ambulatory physical activity of different occupational groups in rural Africa.

The finding that more diverse diets were associated with a lower likelihood of AGR supports previous observations that in countries in earlier stages of nutrition transition, diverse diets are associated with higher consumption of vegetables and fruits [50]. However, diet quality indexes should be used with care because they are based on empirical choices, and location specific food-based dietary guidelines are urgently needed for SSA [49,51].

Integration of targeted screening for AGR in primary care has been widely reviewed [14,52–54], yet evidence on approaches for low income countries is insufficient. Our study demonstrates that because of its simplicity, primary care nurses are able to conduct the FPG test. At a cost of point five three USD per person screened and an average cost of two USD per high risk person detected (using ADA cut-offs), our study demonstrates that screening for AGR among persons aged 35-60 years may be affordable at primary care levels in some low income countries. The average per capita expenditure on health by governments in Africa is twenty-seven USD [55], meaning that some governments in Africa can afford a screening program. Uganda’s government spends only six USD per capita on health, and an addition of 0.53 US per person aged 35-60 years (13.5% of the population) would imply an additional investment of point zero seven USD per capita for early detection of type 2 diabetes. Moreover, the screening interval could be spread out to 3 to 5 yearly. The cost of screening could also be borne by individuals. Uganda’s out-of-pocket expenditure on health is four times the government’s investment [55]. Countries may also screen only people with other existing risk factors (e.g. overweight persons), which approach our study shows is associated with a lower cost.

Limitations of this study include: determining AGR on the basis of a single rapid FPG test, limiting the cost analysis to the health services perspective and to cost-per-person with AGR person found other than cost per QALY gained, possible recall bias in the dietary assessment and not assessing actual quantities of foods eaten. However, FPG is recommended by the WHO as the first line screening test for diabetes and pre-diabetes [12]. Multiple simultaneous tests are of limited value in screening because of their cost implication. With regard to the cost analysis, our study only provides preliminary information and further studies are needed to estimate the longer term benefits of screening with regard to deaths averted and QALYs gained, and to estimate the indirect costs of such a program.

## Conclusions

Because the prevalence of AGR is high among people aged 35-60 years, there is a strong justification for targeted screening of individuals aged 35-60 years when they interface with the health services. The direct medical costs of such screening may be affordable by some countries in SSA. Likewise, diabetes prevention programs could identify high risk groups on the basis of other risk factors (which this study identifies as: obesity, sedentary employment, insufficient physical activity and low dietary diversity). Evidence suggests that risk-based interventions against chronic diseases are cost-effective depending on context [56], but especially where diagnostic tools are lacking [14,19,57].

### Key Messages

- The prevalence of un-detected diabetes and pre-diabetes in rural settings in sub-Saharan Africa is substantial among people aged 35-60 years and calls for preventive action
- There is strong justification for screening of individuals aged 35-60 years in primary care settings, and the associated costs may be affordable to some low income countries in Africa, especially when targeted to people with other risk factors for type 2 diabetes
- Direct lifestyle interventions may also be taken for obese persons, persons with insufficient physical activity, and persons with low dietary diversity

## Supporting Information

Table S1 **Factors associated with diabetes among people aged 35-60 years.**
(DOCX)
