# Supplementary material for: Diabetes and Pre-Diabetes among Persons Aged 35 to 60 Years in Eastern Uganda: Prevalence and Associated Factors
Source: PLoS One. 2013 Aug 14;8(8):e72554. doi: 10.1371/journal.pone.0072554 (PMC3743823; doi:10.1371/journal.pone.0072554)
Supplement: Table S1 — Factors associated with diabetes among people aged 35-60 years. (DOCX) [file pone.0072554.s001.docx]

**Table S1: Factors associated with diabetes among people aged 35-60 years**

| **Factors** | **Category** | **-n-** | **% with diabetes ^a^** | **Unadjusted**  **PRR**  **[95%CI]** | **Adjusted**  **APRR**  **[95%CI] †** | **p-value** |
| --- | --- | --- | --- | --- | --- | --- |
| **Background factors** |  |  |  |  |  |  |
| Sex | Male | 711 | 4.9 | 1.0 |  |  |
|  | Female | 786 | 6.0 | 1.2[0.79-1.86] |  |  |
| Age group | 35-39 years | 457 | 4.0 | 1.0 | 1.0 |  |
|  | 40-49 years | 679 | 5.6 | 1.4[0.81-2.45] | 1.3[0.76-2.32] | 0.321 |
|  | 50-60 years | 361 | 7.2 | 1.9[1.02-3.28] | 1.8[0.98-3.18] | 0.058 |
| Location of residence | Rural | 1275 | 4.9 | 1.0 | 1.0 |  |
|  | Urban | 222 | 8.6 | 1.7[1.06-2.84] | 1.1[0.63-2.10] | 0.644 |
| Education level | None | 291 | 5.2 | 1.0 |  |  |
|  | Primary | 854 | 5.4 | 1.0[0.59-1.84] |  |  |
|  | Secondary | 275 | 5.1 | 1.0[0.49-2.01] |  |  |
|  | Tertiary | 77 | 9.1 | 1.8[0.75-4.17] |  |  |
| Occupation | Subsistence | 933 | 5.5 | 1.0 | 1.0 |  |
|  | Traders | 289 | 5.5 | 1.0[0.58-1.74] | 0.8[0.49-1.45] | 0.542 |
|  | Formal salaried | 98 | 10.2 | 1.9[0.08-3.56] | 1.4[0.76-2.87] | 0.247 |
|  | Mechanic | 177 | 2.8 | 0.5[0.21-1.28] | 0.5[0.18-1.14] | 0.093 |
| SES tertile | Lowest | 495 | 5.7 | 1.0 |  |  |
|  | Middle | 503 | 4.8 | 0.8[0.49-1.43] |  |  |
|  | Highest | 499 | 6.0 | 1.1[0.64-1.75] |  |  |
| Family History of diabetes | No | 1315 | 5.0 | 1.0 | 1.0 |  |
|  | Yes | 182 | 8.8 | 1.8[1.04-2.96] | 1.4[0.82-2.39] | 0.218 |
| BMI | 18.5-24.9 | 988 | 5.0 | 1.0 | 1.0 |  |
|  | <18.5 | 240 | 2.1 | 0.4[0.16-1.04] | 0.4[0.17-1.06] | 0.067 |
|  | 25-29.9 | 189 | 7.9 | 1.6[0.92-2.79] | 1.4[0.78-2.42] | 0.271 |
|  | 30+ | 80 | 16.3 | 3.3[1.93-5.78] | 2.8[1.56-5.08] | 0.001 |
| Hypertensive | No | 1190 | 5.0 | 1.0 |  |  |
|  | Yes | 307 | 7.2 | 1.4[0.88-2.28] |  |  |
| **Socio-behavioural factors** |  |  |  |  |  |  |
| Attains WHO physical activity target | No | 218 | 9.2 | 1.0 | 1.0 |  |
|  | Yes | 1279 | 4.9 | 0.5[0.33-0.86] | 0.7[0.39-1.24] | 0.218 |
| Dietary diversity | Low | 324 | 7.1 | 1.0 | 1.0 |  |
|  | Moderate | 1021 | 5.2 | 0.7[0.46-1.17] | 0.8[0.48-1.28] | 0.335 |
|  | High | 152 | 4.0 | 0.6[0.23-1.34] | 0.6[0.24-1.34] | 0.198 |
| Current tobacco user | No | 1409 | 5.3 | 1.0 |  |  |
|  | Yes | 88 | 8.0 | 1.5[0.71-3.15] |  |  |
| Harmful alcohol use | No | 1427 | 5.7 | 1.0 |  |  |
|  | Yes | 70 | 1.4 | 0.2[0.04-1.78] |  |  |

PRR=Unadjusted Prevalence Rate Ratio; APRR=Adjusted Prevalence Rate Ratio (†Adjusted for residence, age, occupation, family history of diabetes, BMI, physical activity level and dietary diversity).

^a^ Based on World Health Organisation criteria
